# Conversion of marginal land into switchgrass conditionally accrues soil carbon but reduces methane consumption

**DOI:** 10.1038/s41396-021-00916-y

**Published:** 2021-07-01

**Authors:** Colin T. Bates, Arthur Escalas, Jialiang Kuang, Lauren Hale, Yuan Wang, Don Herman, Erin E. Nuccio, Xiaoling Wan, Amrita Bhattacharyya, Ying Fu, Renmao Tian, Gangsheng Wang, Daliang Ning, Yunfeng Yang, Liyou Wu, Jennifer Pett-Ridge, Malay Saha, Kelly Craven, Eoin L. Brodie, Mary Firestone, Jizhong Zhou

**Affiliations:** 1 The Institute for Environmental Genomics, University of Oklahoma, Norman, OK USA; 2 Department of Microbiology and Plant Biology, and School of Civil Engineering and Environmental Sciences, University of Oklahoma, Norman, OK USA; 3 MARBEC, University of Montpellier, Montpellier, France; 4 USDA, Agricultural Research Service, San Joaquin Valley Agricultural Sciences Center, Parlier, CA USA; 5 Noble Research Institute, Ardmore, OK USA; 6 Physical and Life Sciences Directorate, Lawrence Livermore National Laboratory, Livermore, CA USA; 7 Department of Environmental Science, Policy and Management, University of California, Berkeley, CA USA; 8 The Key Laboratory of Aquatic Biodiversity and Conservation of the Chinese Academy of Sciences; Institute of Hydrobiology, Chinese Academy of Sciences, Wuhan, China; 9 Earth and Environmental Sciences Area, Lawrence Berkeley National Laboratory, Berkeley, CA USA; 10 State Key Joint Laboratory of Environment Simulation and Pollution Control, School of Environment, Tsinghua University, Beijing, China

**Keywords:** Grassland ecology, Biogeochemistry, Microbial communities, Plant ecology

## Abstract

Switchgrass is a deep-rooted perennial native to the US prairies and an attractive feedstock for bioenergy production; when cultivated on marginal soils it can provide a potential mechanism to sequester and accumulate soil carbon (C). However, the impacts of switchgrass establishment on soil biotic/abiotic properties are poorly understood. Additionally, few studies have reported the effects of switchgrass cultivation on marginal lands that have low soil nutrient quality (N/P) or in areas that have experienced high rates of soil erosion. Here, we report a comparative analyses of soil greenhouse gases (GHG), soil chemistry, and microbial communities in two contrasting soil types (with or without switchgrass) over 17 months (1428 soil samples). These soils are highly eroded, ‘Dust Bowl’ remnant field sites in southern Oklahoma, USA. Our results revealed that soil C significantly increased at the sandy-loam (SL) site, but not at the clay-loam (CL) site. Significantly higher CO_2_ flux was observed from the CL switchgrass site, along with reduced microbial diversity (both alpha and beta). Strikingly, methane (CH_4_) consumption was significantly reduced by an estimated 39 and 47% at the SL and CL switchgrass sites, respectively. Together, our results suggest that soil C stocks and GHG fluxes are distinctly different at highly degraded sites when switchgrass has been cultivated, implying that carbon balance considerations should be accounted for to fully evaluate the sustainability of deep-rooted perennial grass cultivation in marginal lands.

## Introduction

Taking place over three waves during the 1930s, the American ‘Dust Bowl’ was a catastrophic ecological disaster that brought severe drought and dust storms to the central prairies of the US and affected roughly 40 million hectares of land [1–3]. These climatic events, combined with many years of poor land management and soil cultivation, exacerbated topsoil erosion, creating many ‘marginal’ lands of low soil nutrient quality, notably across Oklahoma and the Southwestern USA (Texas, Kansas, Colorado, and New Mexico). Since then, many of these sites have remained suboptimal for agricultural development. It has been suggested that widespread cultivation of deep-rooted perennial grasses may aid in soil restoration at these sites, while also offering further economic benefits to farmers in the form of cellulosic feedstocks for bioenergy production [4]. It is estimated that 15 million hectares of arable land would need to be converted into biofuel crops to meet the US Department of Energy’s plan to replace 30% of transportation fossil fuels with biofuels by 2030 [5, 6]. An estimated 11% of the contiguous USA is considered nutrient-poor or ‘marginal’ land [7] and currently represents an under-utilized resource that may be well suited for cultivation of switchgrass or other deep-rooted perennials [8].

Switchgrass (*Panicum virgatum* L.), a tall perennial deep-rooted grass native to the Central North American Plains, is a well-studied bioenergy crop, and thought to be suitable for large-scale cultivation in the USA [9]. Switchgrass may even be implemented more broadly as it has been projected to grow favorably in numerous regions globally, both with and without irrigation [10]. This enthusiasm stems from switchgrass’ high productivity even on low-quality soils unfit for traditional row-crop agriculture, with little to no additional inputs [11]. Long-term cultivation experiments suggest that switchgrass can provide a net input of C into soil [4, 12, 13]. Therefore, large-scale switchgrass cultivation may help to simultaneously offset GHG emissions and improve soil quality through C sequestration at nutrient-poor sites [14]. Switchgrass is also known to be highly drought tolerant [15] and can prevent topsoil erosion owing to its high root biomass, which increases the surface area for exudation, and can further improve soil C stability and aggregate formation [16, 17]. Like other perennial crops, switchgrass has been broadly associated with increases in soil C at many experimental sites across the central Great American Plains [18–20]. However, only a few studies have evaluated switchgrass cultivation at sites with low soil N, C, or P contents or in marginal lands that have experienced high rates of topsoil erosion [4, 21]. Thus, we currently have a very limited understanding of how switchgrass row-crop systems in nutrient-poor marginal lands can affect (i) soil geochemical composition, (ii) soil microbial diversity, and (iii) overall ecosystem functionality, specifically GHG fluxes.

Potential C accrual due to increased root inputs may be offset by higher soil CO_2_ production arising from stimulated microbial C mineralization, the so-called ‘rhizosphere priming’ effect [22]. Thus, while the total amount of soil organic carbon (SOC) may increase, the C input by switchgrass along a depth profile may also prime the degradation of preexisting SOC by the indigenous soil microbial community [23, 24]. Regulation of this priming response depends on the mineral composition [25], nutrient content [26], and microorganism present [27]. Therefore, to evaluate the carbon cycle benefits of deep-rooted perennials in marginal lands, it is essential to assess not only the balance of C-based GHG fluxes (CO_2_ and CH_4_) but also other GHG (such as N_2_O) and changes in soil microbiomes.

Because soil microorganisms are critical drivers of soil nutrient cycling, understanding plant–microbe interactions during switchgrass cultivation could inform land management strategies that promote soil nutrient acquisition (e.g., nitrogen fixation) and recycling, while reducing GHG emissions. A recent review of switchgrass-associated microbiomes suggests that mycorrhizal fungi, associated N-fixing bacteria, and fungal endophytes play particularly key roles in increasing switchgrass biomass, providing a substantial portion of the plant’s nitrogen demand, and improving drought tolerance [28]. By examining the microbial diversity and associated functional processes of switchgrass-influenced systems, prior research has revealed some of the mechanisms underlying the enhancement of ecosystem services such as C sequestration, soil fertility, and regulation of GHG emissions [5, 29–34]. For instance, it has been shown that N fertilization, at least at some sites, did not increase soil-surface carbon dioxide (CO_2_) emissions despite promoting above- [35, 36] and below-ground biomass [37]. However, the relative impact of methane (CH_4_) emissions during switchgrass establishment at highly eroded sites is not yet fully understood [38–40]. Additionally, the ecological consequences of land conversion, its impact on soil microbial diversity, and functionality, as well as the overall sustainability of switchgrass cultivation in low-quality soils, remain to be demonstrated.

In grasslands and agricultural systems, above (i.e., plant) and below (i.e., microbial) ground biodiversity [41] and biomass [42, 43] are related, and edaphic conditions can influence these relationships. A meta-analysis of temperate grasslands suggests a correlation between plant and bacterial beta diversity but not alpha diversity [44]. Previous studies of the microbiomes of monoculture agroecosystems have revealed differences in nitrogen-fixing bacterial communities [45], seasonal dynamics [46], and core microbiome members between different plant species [47]. Therefore, in the transition from an annual plant species to a monoculture perennial crop, we expect a decrease of both alpha and beta microbial diversity.

In this study, we monitored the below-ground microbiological and chemical impacts of switchgrass establishment and consequences for soil GHG emissions over two consecutive growing seasons (*n* = 17 months) in two nutrient-poor (N and P) field sites with low C content in southern Oklahoma (designated SL and CL for their sandy-loam soil and clay-loam soil texture, respectively). We compared soil belowground (root) productivity, chemistry (C, N, and P), soil GHG fluxes (CO_2_, CH_4_, and N_2_O), and microbial community composition at each site with switchgrass or without switchgrass (natural fallow plots). We hypothesize that the following trends would be observed in the field plots with switchgrass: (i) increased topsoil C concentrations over time; (ii) CO_2_ production increases, with CH_4_ emission and N_2_O fluxes remaining relatively stable; and (iii) the microbial community would be altered during establishment and both species richness and beta diversity would decrease over time.

## Methods

### Field site, soil sampling, and root biomass estimation

Samples were collected from two sites in southern Oklahoma, a SL site near the Texas border (33.881715°N, −97.275167°W) and a CL site in Ardmore (34.172100°N, −97.07953°W) (Table S1). Prior to our experiment, the SL field site experienced crop rotation between small grains (wheat, rye, oat, and triticale) in the winter season and soybeans in the summer. Soybean was terminated before seed set to prepare the land for small grain planting as a cover crop. At the CL site, Bermuda grass was the dominant plant cover for at least 20 years prior to our study. These sites were selected for their low soil nutrient content (N and P) and their history of topsoil erosion.

In the summer of 2016, two plots were established at each site, a switchgrass field plot (27 × 22 m) containing 500 genetically distinct individuals of the lowland Alamo variety with a 1 m spacing between plants and a corresponding fallow plot (27 × 22 m) (Fig. S1a, b). All plots were tilled to 30 cm before the start of the experiment, and then planted with switchgrass seedlings with 1 m spacing. Switchgrass plots were sustainably managed, without any chemical fertilizers, herbicides, or watering. Fallow plots were allowed to undergo a natural succession of grasses and weeds over the time course of the experiment and served as controls to compare the dynamics in soil carbon, trace gas fluxes, and microbial community composition with the switchgrass treatment. In November 2016, a survey was conducted to determine the vegetative cover of the two fallow plots. The SL fallow surface was mostly composed of bare soil (~52%); plant litter (~31%) and annual forbs (~17%) covered the rest of the plot. The CL fallow surface was dominated by annual grass species (~86%), annual forbs (~2%) and bare soil (~1%) covering the remaining surface of the plot. Common plant genera at both fallow sites included the following: *Oxalis, Dichanthelium, Cynodon, Brassica, Lamium, Trifolium, Cyperus, Geranium, Erigeron, Conyza*, and *Digitaria*.

At each plot, to allow GHG measurements, 21 PVC collars (diameter 23.63 × 12.8 cm height) (Fig. S1c) were embedded 8 cm into the soil and placed in a cross design across the field with five collars extending in each cardinal direction from a central origin collar at the plot center. After trace gas measurement from each collar, two soil cores (0–20 cm in depth) were taken from within a 20 cm radius of each collar (Fig. S1d), thoroughly mixed, and separated into two aliquots, one for geochemical analyses and one for DNA extraction. Sampling flags were placed to prevent re-sampling the same location twice, and each core was filled by topsoil taken from outside the plot. All soil samples were immediately stored on dry ice, transported back to the lab in less than 5 h, and stored at either 5 °C for geochemical analyses or −80 °C for DNA extraction.

In May 2017, the total belowground root biomass was estimated using a method accounting for differences in root density between the plants in each row [48]. Briefly, six pairs of plants were selected, and a transect consisting of four 0–1 m cores was performed between each pair of plants (Fig. S2a). The two cores at the end of the transect represent the root density ‘with-in’ rows, and the two middle cores represent the root density of the ‘between-rows’. Each core was divided into five 20 cm ‘slices’ of soil. For each depth, roots were collected by sieving and soaking the soil in water, before being dried and weighted. For fallow plots, four randomly assigned 1 m^2^ subplots were selected, in which a four 0–1 m soil cores transect was collected (Fig. S2b). No roots were detected from fallow soil cores below 60 cm depth.

### Soil geochemistry, pH, and moisture

Soil pH, moisture, total soil C and N, plant-available P, nitrate (NO_3_), and ammonium (NH_4_) pools were measured according to standard methods [49]. Briefly, 10 g of soil was placed into a 50 ml tube with distilled H_2_O added to the 50 ml fill line. Tubes were gently shaken for 30 min and given an hour to settle before pH measurement using a pH probe (Acccumet excel XL15 pH meter, Fisher Scientific, Hampton NH, USA). Soil moisture was determined by a gravimetric drying protocol that dried > 5 g of soil for one week at > 60 °C before re-weighing to establish the percent of water lost. To determine other soil geochemical parameters, soil samples were dried in an oven at 60 °C for a week followed by sieving to remove unwanted material with a 4 mm sieve. Soil samples were then shipped seasonally to the Oklahoma State University (OSU) soil testing lab where Mehlich III extractions (to quantify the plant available P in the soil) and KCL extractions (to determine NH_4_ and NO_3_ concentrations) were performed and total soil C/N amounts were measured via dry combustion (LECO corporation, St. Joseph MI, USA). To establish initial conditions, 3 replicate 2-meter soil pits were dug at each site prior to switchgrass planting, and sampled every 2 cm (Table S1). Soil chemical analyses were conducted on air-dried soils at the Oregon State University Central Analytical Laboratory in Corvallis OR. Particle size analysis (sand/silt/clay) was conducted on 40 g of soil using the hydrometer method [50]. pH and electrical conductivity (EC) were determined in a 1:1 soil/water ratio [51]. Total C and N were determined by combustion and a thermal conductivity detector [52]. Loss on ignition organic matter was measured after treatment in a muffle furnace [53]. Total P content was determined by digestion with nitric acid; extracts were analyzed by Inductively Coupled Plasma Atomic Emission Spectrometry (ICP-AES) [54]. Bioavailable P was determined using the Bray and Olsen methods for acidic to neutral pH soils [55–57]. Organic P was measured on 2 g soil extracted with NaOH-EDTA [58], amorphous P by ammonium-oxalate extraction [59], and crystalline P by citrate-dithionite extraction [60].

### Environmental parameters

Daily environmental data for 21 different environmental variables (at 5 to 15-min resolution) were obtained from two weather monitoring stations of the Oklahoma MESONET network (http://mesonet.org/) closest to the field sites (Ardmore and Burneyville, located 1.43 km and 2.3 km from CL and SL, respectively). Variables used included air temperature, bare soil temperature, covered soil temperature, atmospheric pressure, relative humidity, and precipitation (Tables S2 and S3).

### Trace gas fluxes

CO_2_, CH_4_, and N_2_O fluxes were measured monthly via cavity ring-down spectrometry using a Picarro G2508 analyzer (Picarro, Santa Clara, CA, U.S.A.). Measurements were taken continuously every 2 s from a total of 6 min per collar, to obtain gas concentrations in parts per million. Raw data from each gas were separated and then manually inspected to remove the beginning and the end of the measurements, which are often influenced by the pushing/pulling of the gas chamber (volume of the chamber + collar = 7917 cm^3^). Then three models (linear, quadratic, and exponential) were fitted for each sample and gas species to characterize the variation of gas concentrations across time and the ‘best model’ was selected based on AIC scores. Flux estimations for each of the gases were then calculated using the following equation [61]:

$$F = \frac{{dc}}{{dt}} \cdot \frac{{PV}}{{A \cdot R(273.15 + T)}} \times 3600$$

Where $\frac{{dc}}{{dt}}$ is the slope of the best fit model at *t* = 0, *V* is the chamber volume (L), *A* is the chamber area (m^2^), *R* is the gas constant in L atm K^−1^ mol^−1^, and *T* is the temperature in Celsius, when the chamber pressure is assumed to be equal to 1 atm. The 3600 factor is included to convert the flux to hourly values. For CO_2_ fluxes, *F* was then divided by 1000 to obtain the units of millimoles per m^2^ per hour.

### Soil DNA extractions, microbial community sequencing and analysis

All molecular biology procedures and DNA sequencing were performed at the Institute for Environmental Genomics (IEG, University of Oklahoma, USA). A freeze grinding method [62] was combined with the Powersoil DNA extraction kit (Qiagen, Venlo, Netherlands) to extract DNA from a total of 1428 soil samples, which yielded soil DNA of both high quantity and quality. For microbial community profiling, a two-step PCR method [63] was used for amplification of the V4 region of the bacterial 16 S rRNA gene using the 515 F, 5′-GTGCCAGCMGCCGCGGTAA-3′ and 806 R, 5′-GGACTACHVGGGTWTCTAAT-3′ primers. Sequencing of the 16 S rRNA gene amplicons was conducted on the Illumina Mi-Seq DNA sequencing platform (Illumina Inc., San Diego, CA, U.S.A.). Amplicon sequence data were analyzed using an internal pipeline known as the Amplicon Sequencing Analysis Pipeline [64] (ASAP, version 1.4). MiSeq sequences were quality checked with FastQC (version 0.11.5), pair-end sequences were merged based on their 3′ overlap using PEAR (version 0.9.10) with a quality score cutoff set to 20, and assembly length between 200–400 with the minimum overlap length set to 50 bp. The program *split_libraries_fastq.py* from the QIIME package [65] (version 1.9.1) was used to assign reads to each sample (demultiplexing) based on the barcodes for each individual sample with a maximum allowed barcode error of 0 and the trimming quality score set to 20. Primer sequences were then trimmed and removed. Sequences from multiple split libraries were merged. Dereplication was performed by USEARCH [66] (version 9.2.64) using the command *fastx_uniques* (utilizing the size-out option for sequence abundance output). Operational Taxonomic Units (OTUs) were clustered using UPARSE, with the OTU identity threshold set to 0.97 and the singletons/chimeric sequences removed [67]. An OTU table was generated by the command *-usearch_global* in USEARCH. Each representative sequence for each OTU was classified with the RDP Classifier [68] (16 S: training set 16, June 2016) with the confidence cutoff set to 0.8. OTUs in the 16 S sequence reads assigned to Chloroplast at the Order level were removed. Representative sequences for each OTU were used to construct a phylogenetic tree. Sequences were then aligned using MAFFT [69] (version 3.8.31) and alignments were filtered using Gblocks [70] (version 0.91b) with the options -t=d, -b4=3 and -b5=h. FastTree [71] was used for constructing the phylogenetic tree using the filtered alignments. The phylogenetic tree and OTU tables were used to calculate alpha diversity (phylogenetic based indexes) and beta diversity (UniFrac distance) using programs packaged in QIIME [72] and R [73].

### Statistical analyses

All analyses were conducted using R statistical software [73] (3.4.4) and figures were produced using the package ggplot2 [74]. Data normality was tested using the Shapiro test. We tested for differences in GHG flux and microbial alpha diversity between plots using linear mixed models to correct for repeated measurements (i.e., collars within plots) and to analyze the data over time (R package *lme4*) [75]. Pairwise comparisons for soil trace gas production between treatments were conducted using Wilcoxon Rank Sum test and effect sizes were calculated using Mann–Whitney U Test. Differences in soil biogeochemical properties between treatment were tested using Kruskal–Wallis test and effect size was calculated using epsilon squared. Soil geochemical dissimilarity was calculated from scaled data using Euclidean distances (*vegan* R package). Then mean dissimilarity across GHG collars was used to construct linear mixed models to view changes in dissimilarity over time. Differences in microbial community structure across plot, site, and time were tested using a PERMANOVA test based on Bray Curtis and weighted-UniFrac dissimilarity for taxonomic and phylogenetic diversity, respectively. We used PERMDISP [76], a distance-based test for homogeneity of multivariate dispersion, to assess differences in beta diversity between treatments using the ‘betadisper’ function (*vegan* R package). Differences in relative abundance between groups and time points were calculated by multiple Student’s *t* tests; *p*-values were adjusted by conservative Bonferroni correction to compensate for increased Type 1 errors over multiple time points.

Structural equation modeling (SEM) was used to explore the direct and indirect relationships among environmental variables and GHG fluxes (CO_2_ and CH_4_) at both sites. Based on correlation analyses among all variables (Fig. S3), we first considered a full model that included all reasonable pathways, then eliminated nonsignificant pathways until we obtained a final model with only significant pathways. We used a χ^2^ test and the root mean square error (RMSE) to evaluate the fit of our model. To correct for potential temporal autocorrelation, we used averaged microbial and environmental data for each trace-gas collar sampling location, averaged across time points within each plot. For the CO_2_ model, all sampling locations were pooled in a single SEM model (*n* = 84). However, for CH_4_, a consistent SG effect was found across the sites. Therefore, two models (*n* = 42) were created, one for the SG and one for FL plots to compare and identify differences in environmental factors that contribute to the CH_4_ fluxes between the two treatments. The SEM-related analysis was performed using the lavaan R package [77].

## Results

### Changes in root biomass and soil chemistry

**Fig. 1:**
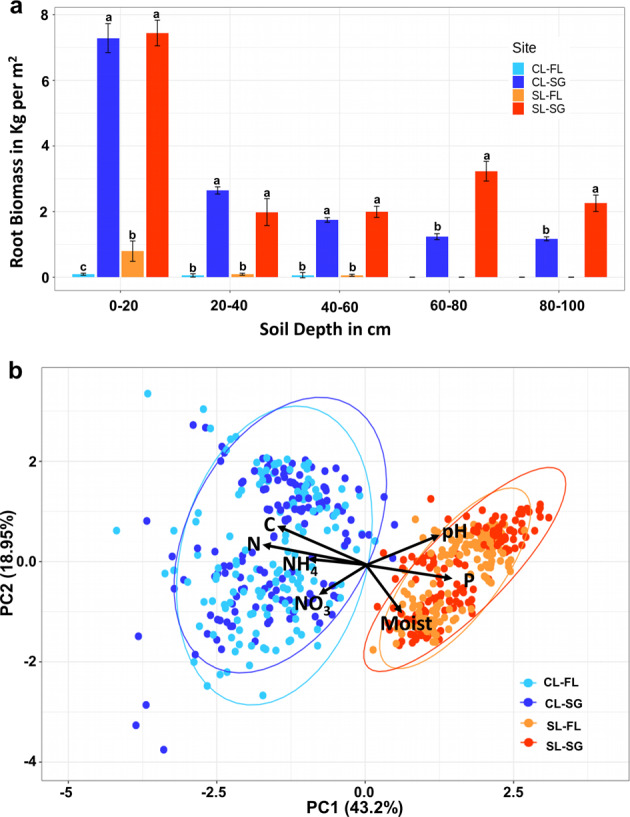
Differences in root biomass estimates and soil chemical properties between the two studied sites. **a** Difference between fallow and switchgrass plots for estimated root biomass by depths (*n* = 4), letters indicate significant difference between groups by Student’s *t* test; **b** Principal component analysis of soil chemical properties by site and treatment (seasonal samples displayed, *n* = 588). Blue colors represent the CL site while red/orange colors signify the SL site. Dark colors represent the SG samples. Variation contained in each PC axis are displayed next to each axis.

We observed a large difference in the field-scale estimates of belowground root biomass (Fig. 1a) between the switchgrass and fallow plots (17.8 and 64 times higher for SL and CL, respectively). Root biomass was estimated for each soil layer in kilograms per meter squared. The estimated total root biomass of all soil layers in the top 1 m added together for the switchgrass plots was 16.9 kg/m^2^ for the SL site and 14.1 kg/m^2^ for the CL site, while the fallow plots had 0.95 kg/m^2^ of roots in the SL and 0.22 kg/m^2^ in the CL site. Generally, root biomass decreased along the soil depth at both sites. The SL switchgrass site had higher root biomass estimates at lower depths (60–100 cm, *p* value < 0.05 by Student’s *t* test) than the CL site, which contributed to a slightly higher total root biomass.

A principal component analysis (PCA) of the soil chemistry data (Soil C, N, P, NO_3_, NH_4_, pH, and soil moisture levels) revealed strong differences between the two sites (Fig. 1b). Heterogeneity of the geochemical parameters was higher at the CL site with an average dissimilarity of 2.96 ± 0.73 compared with 2.52 ± 0.24 for the SL site, and illustrated by the greater dispersion of samples from the CL site in Fig. 1b.

**Fig. 2:**
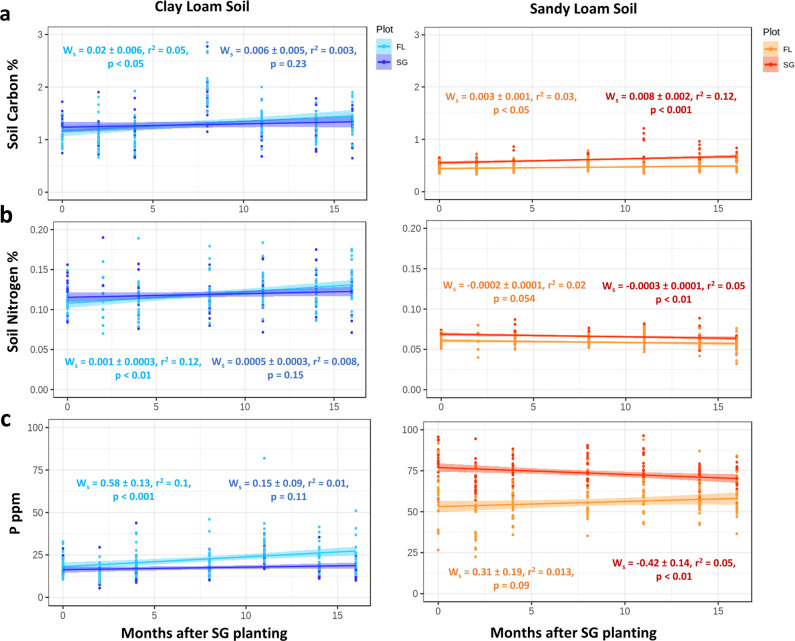
Changes in soil chemistry through two seasons of switchgrass establishment. **a** Total soil carbon percentages. **b** Total soil nitrogen percentages. **c** Concentration of plant available phosphate content in parts per million. The best linear model describing the relationship is presented. W_s_: estimated model slope and associated error. *p*-values represent the significance of each model. Each time point is comprised of twenty-one replicates per plot (*n* = 588, seasonal samples).

The total soil surface C (0–20 cm) at the SL site increased over the 17-month period in the switchgrass plot (Fig. 2a) (*r*^2^ = 0.12, *p* < 0.001) and was significantly higher than in the fallow plot (Table 1, *p* < 0.001, large effect size = 0.4). Switchgrass also had a homogenizing effect for soil C, probably due to the increase in belowground root biomass, and reduced the overall dissimilarity between samples compared to the fallow plots which had patchy plant cover. These increases in soil C occurred evenly across the plot area (Fig. S4a). In contrast, the total soil C content remained constant in the CL switchgrass plot (Fig. 2a).

**Table 1:** **Differences in physico-chemical soil properties for each site and treatment after 17 months**.

Variable	Silt loam fallow	Silt loam switchgrass	Kruskal–Wallis tests			Clay loam fallow	Clay loam switchgrass	Kruskal–Wallis tests		
	Mean ± SD	Mean ± SD	Chi-squared	*p*	Effect size	Mean ± SD	Mean ± SD	Chi-squared	*p*	Effect size
pH	6.5 ± 0.67	6.7 ± 0.95	1.32	0.25	-	5.73 ± 0.44	5.85 ± 0.57	2.17	0.14	-
Soil moisture (%)	7.1 ± 4.1	8.7 ± 6	3.27	0.07	-	10.4 ± 6.8	9.82 ± 5.5	0.75	0.39	-
Total soil C (%)	0.47 ± 0.09	0.61 ± 0.12	118	< 0.0001***	0.40*** Large	1.3 ± 0.42	1.3 ± 0.36	0.86	0.40	-
Total soil N (%)	0.06 ± 0.01	0.07 ± 0.01	55.4	< 0.0001***	0.19** Medium	0.11 ± 0.02	0.11 ± 0.02	0.27	0.6	-
Phosphorus (ppm)	55.6 ± 13	73.7 ± 9.7	128	< 0.0001***	0.44*** Large	22.6 ± 9.7	17.5 ± 6.5	27.7	< 0.001**	0.095** Medium
Nitrate (ppm)	3.5 ± 3.7	2.2 ± 3	16.8	< 0.001**	0.06* Small	8.3 ± 9.8	8.9 ± 15	2.37	0.12	-
Ammonium (ppm)	13.6 ± 11	14.5 ± 10	1.73	0.19	-	18.5 ± 11	17.9 ± 11.5	0.61	0.44	-

Values are mean ± SD values and significance was tested by Kruskal–Wallis rank sum test (*n* = 588). Astreriks (*) indicate significance of *p*-values.

*Effect size shown by epsilon squared with *small (0.01–<0.08), **medium (0.08–<0.26), and ***large (≥0.26) ranges.

Total surface soil N was significantly higher in the SL switchgrass plot compared to the fallow plot (Table 1, *p* < 0.0001, medium effect size = 0.19) and these N levels significantly decreased over time (*r*^2^ = 0.05, *p* < 0.01) (Fig. 2b), coinciding with an increase in the soil N heterogeneity in the plot (*r*^2^ = 0.12, *p* < 0.0001) (Fig. S4b). In contrast, we measured a significant increase in the total soil N in the CL fallow plot (*r*^2^ = 0.12, *p* < 0.01) (Fig. 2b). Nitrate concentration were significantly reduced for the switchgrass treatment at the SL site (Table 1, *p* < 0.001, small effect size = 0.06). All sites and plots showed a significant reduction in NO_3_ concentrations over time (Fig. S5a) along with increased homogeneity. No significant differences were observed in soil NH_4_^+^ concentrations during the length of our study at either site (Fig. S5b). Total plant available P levels decreased over time in the SL site (*r*^2^ = 0.05, *p* < 0.01) (Fig. 2c) and became more homogeneous across the plot despite the SL switchgrass treatment having significantly higher total plant available P content compared to the fallow (Table 1, *p* < 0.0001, large effect size = 0.44). In the CL site, plant available P also decreased in the switchgrass plot compared to the fallow (Table 1, *p* < 0.001, medium effect size = 0.095, and Fig. 2c).

### Greenhouse gas (GHG) fluxes at the soil–atmosphere interface

**Fig. 3:**
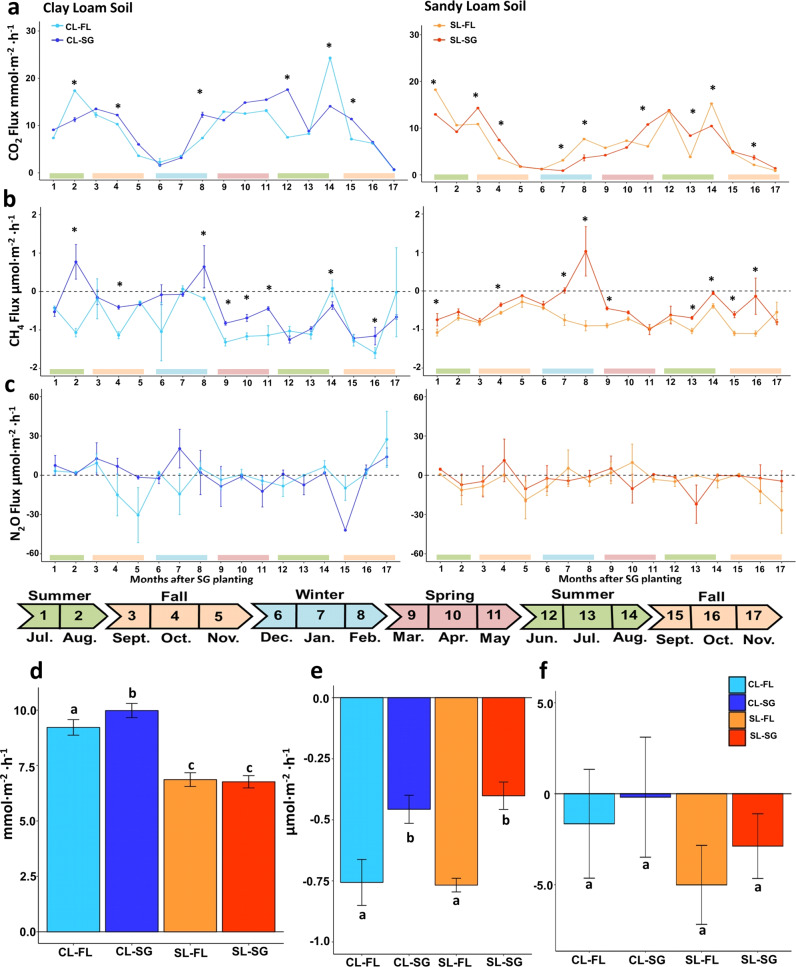
Greenhouse gas (GHG) fluxes during grassland conversion to switchgrass. **a**, **b**, **c** GHG fluxes at each site over 17 months (mean and standard error estimated using 21 replicates for each time points, *n* = 1428) for: **a** carbon dioxide flux; **b** methane flux; **c** nitrous oxide. **d** Average GHG fluxes over 17 months for **d** carbon dioxide; **e** methane flux; **f** nitrous oxide flux. Different letters and asterisk indicate significant difference between groups by Wilcoxon sign test with *p*-value < 0.01.

CO_2_ flux exhibited a similar seasonal trend at both sites with the apex of emissions occurring during summer months and the minimum in late Fall/early Winter months (Fig. 3). At the SL site, switchgrass treatment led to significantly higher total CO_2_ flux for 29% of the months after switchgrass planting (Wilcoxon *p* < 0.001, Fig. 3a) while the fallow was significantly higher for only 24% of the total months measured. The average CO_2_ flux over the 17 months did not differ in the SL site between switchgrass (6.76 ± 5.23 millimoles m^2^ h^−1^) and the fallow plots (6.87 ± 5.87 millimoles m^2^ h^−1^). At the CL site, there was a significant difference between plot treatments in the average CO_2_ flux over the 17-month period (*p* < 0.001) with the switchgrass plot at 9.98 ± 6.04 millimoles m^2^ h^−1^ and the fallow at 9.22 ± 6.62 millimoles m^2^ h^−1^, although the effect size was small (0.13). When comparing the two sites, the CL site had significantly higher total soil CO_2_ fluxes for both switchgrass and fallow plots than those measured at SL (Wilcoxon *p* < 0.001).

CH_4_ fluxes (Fig. 3b, Table S4) differed significantly between switchgrass and fallow (Wilcoxon *p* < 0.001, small effect size = 0.15), with a tendency toward higher CH_4_ emissions or lower CH_4_ consumption levels in the switchgrass plots for 41% of the months after switchgrass was planted (41 and 52% for CL and SL, respectively). CH_4_ flux in the CL fallow was higher only at one time point (14^th^ month after switchgrass establishment). Overall, the 17-month average CH_4_ consumption rate was −0.44 ± 1.07 micromoles m^2^ h^−1^ for switchgrass treatments (−0.46 ± 1.08 and −0.41 ± 1.06 micromoles m^2^ h^−1^ for CL and SL, respectively) and −0.77 ± 1.15 in the fallow (−0.76 ± 1.78 and −0.77 ± 0.53 micromoles m^2^ h^−1^ for CL and SL, respectively) (Fig. 3e, Table S4). Taken together, we observed a significant effect of switchgrass cultivation, with reduced CH_4_ consumption rates at both sites (*p* < 0.05, a small effect size = 0.14).

We did not measure significant differences for N_2_O fluxes between the switchgrass (−0.26 ± 2.55 micromoles m^2^ h^−1^ at CL and −2.88 ± 2.09 micromoles m^2^ h^−1^ at SL) and fallow plots (−1.65 ± 2.5 micromoles m^−2^ h^−1^ at CL and −5.01 ± 2.16 micromoles m^−2^ h^−1^ at SL) at either site over the 17 months of observations (Fig. 3f).

### Microbial community dynamics

**Fig. 4:**
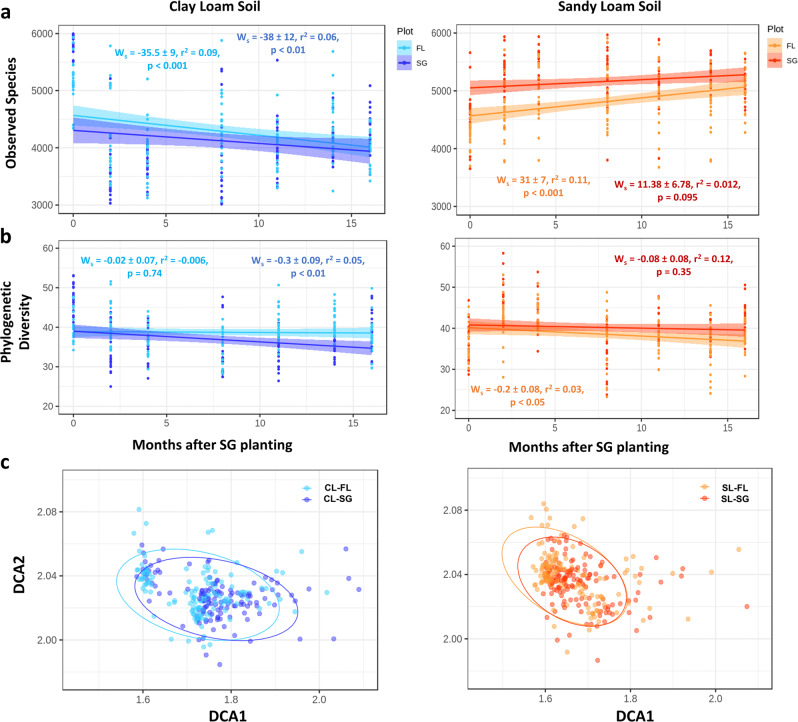
Changes in microbial diversity and structure in response to switchgrass planting. **a** Number of observed species through time. **b** Phylogenetic diversity. **c** Detrended correspondence analysis of the 16 S community separated by site for all time points and plots (*n* = 1428). Significant differences were found between sites, plant cover types, and through time (PERMANOVA, *p* < 0.01). Dark colors represent the switchgrass samples.

Microbial alpha diversity, calculated as OTU richness, showed a site-specific response to switchgrass cultivation. In the SL site, OTU richness was significantly higher in the switchgrass plot (Table S4, *p* < 0.0001, medium effect size = 0.38). OTU richness did not change over time in the SL switchgrass plot (Fig. 4a) but increased in the fallow plot (*p* < 0.001), despite a decrease in phylogenetic diversity (PD) (*p* < 0.05, Fig. 4b). At the CL site, microbial species richness decreased significantly over time in both switchgrass (*p* < 0.01) and fallow plots (*p* < 0.001). For PD, this decay was observed only in the switchgrass plot (*p* < 0.01). Chao1 and Shannon indices showed similar trends per site over time (Fig. S6). Switchgrass cultivation significantly (PERMDISP, *p* < 0.05) decreased the beta diversity at the CL (FL = 0.2323 and SG = 0.2179) and SL (FL = 0.2349 and SG = 0.2273) sites when compared to the paired fallow plots, with decreases in the average distance to the median for the homogeneity of multivariate dispersion.

**Fig. 5:**
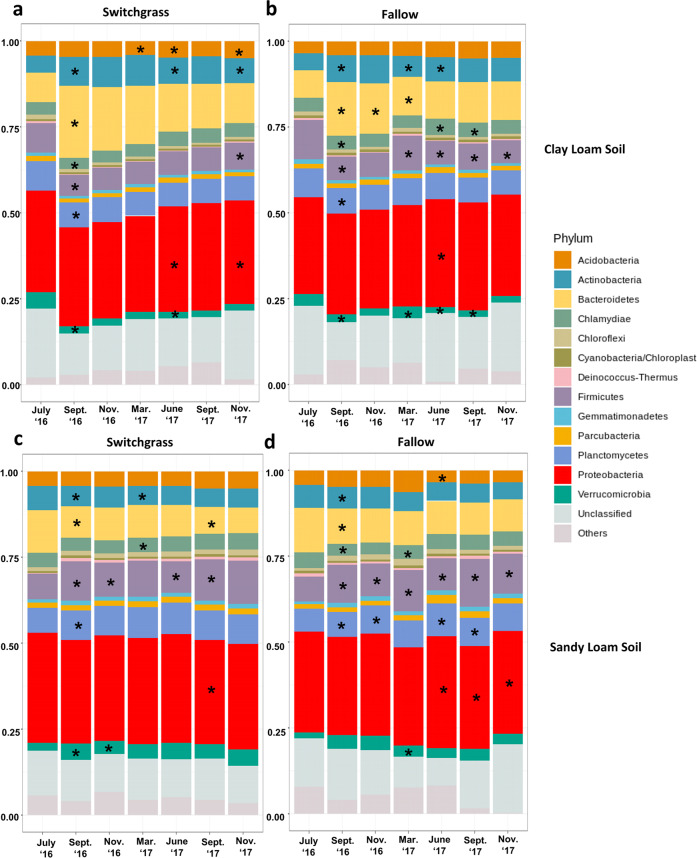
Changes of relative abundance for major phyla. Taxonomic identity was determined with the RDP classifier at 80% sequence match criteria. OTU table was trimmed by abundant OTUs (>0.001%). Difference between time points within each plot for: **a** Clay-loam switchgrass (CL-SG) plot; **b** Clay-loam fallow (CL-FL) plot; **c** Sandy-loam switchgrass (SL-SG) plot; **d** Sandy-loam fallow (SL-FL) plot. Significant differences between the previous time point for each group denoted by asterisk (*) symbols within each phyla bar.

We observed significant differences in the bacterial community structure (beta diversity) between sites, plant cover type, and over time (Fig. 4c, PERMANOVA, *p* < 0.01, Table S5). Relative abundance of major phyla showed large changes from the initial planting and two months after the experiment began (Fig. 5). At all sites, at least five abundant phyla exhibited changes in relative abundance. The relative abundance of Firmicutes (0.6–0.14%) changed over the course of the experiment in both fallow plots. The structure of microbial communities from the switchgrass plots appeared less variable than in the corresponding fallow plots. In the CL site, the strongest differences in dominant phyla relative abundance between plots (switchgrass vs fallow) were observed at eight and fourteen months after switchgrass planting (February 2017 and August 2017, Table S6). After eight months, seven phyla (Actinobacteria, Bacteroidetes, Chloroflexi, Deinococcus-Thermus, Firmicutes, Planctomycetes, and Verrucomicrobia) exhibited different abundance between treatment, while only four phyla (Actinobacteria, Chloroflexi, Cyanobacteria, and Deinococcus-Thermus) were different after 14 months. For the SL site, the largest shifts in community composition occurred in the last two time points, i.e., 14 and 16 months after switchgrass establishment. After 14 months, three phyla were significantly different between treatment (Acidobacteria, Bacteroidetes, and Deinococcus-Thermus) and after 16 months four groups were significantly different (Acidobacteria, Bacteroidetes, Cyanobacteria, and Verrucomicrobia).

**Fig. 6:**
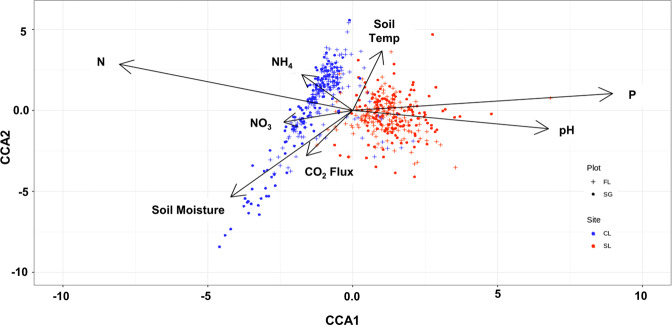
Relationships between environmental factors and microbial communities structure. Canonical correspondence analysis (CCA) linking microbial communities structure with environmental variables (*n* = 1428). Samples are shown by plot and site type with significant environmental variables shown in black arrows.

We used canonical correspondence analysis (CCA) to link environmental variables to the microbial community (Fig. 6). A clear separation between microbial communities from the two sites was observed. Microbial communities from the SL site were correlated with plant available P and soil pH, while CL communities were associated with total soil N, NH_4_, and NO_3_. In addition, fallow communities from CL were far more dispersed, with switchgrass soil communities at this site clustered by N source or along a soil moisture gradient.

### Structural equation model links microbial features to soil properties and trace gas fluxes

**Fig. 7:**
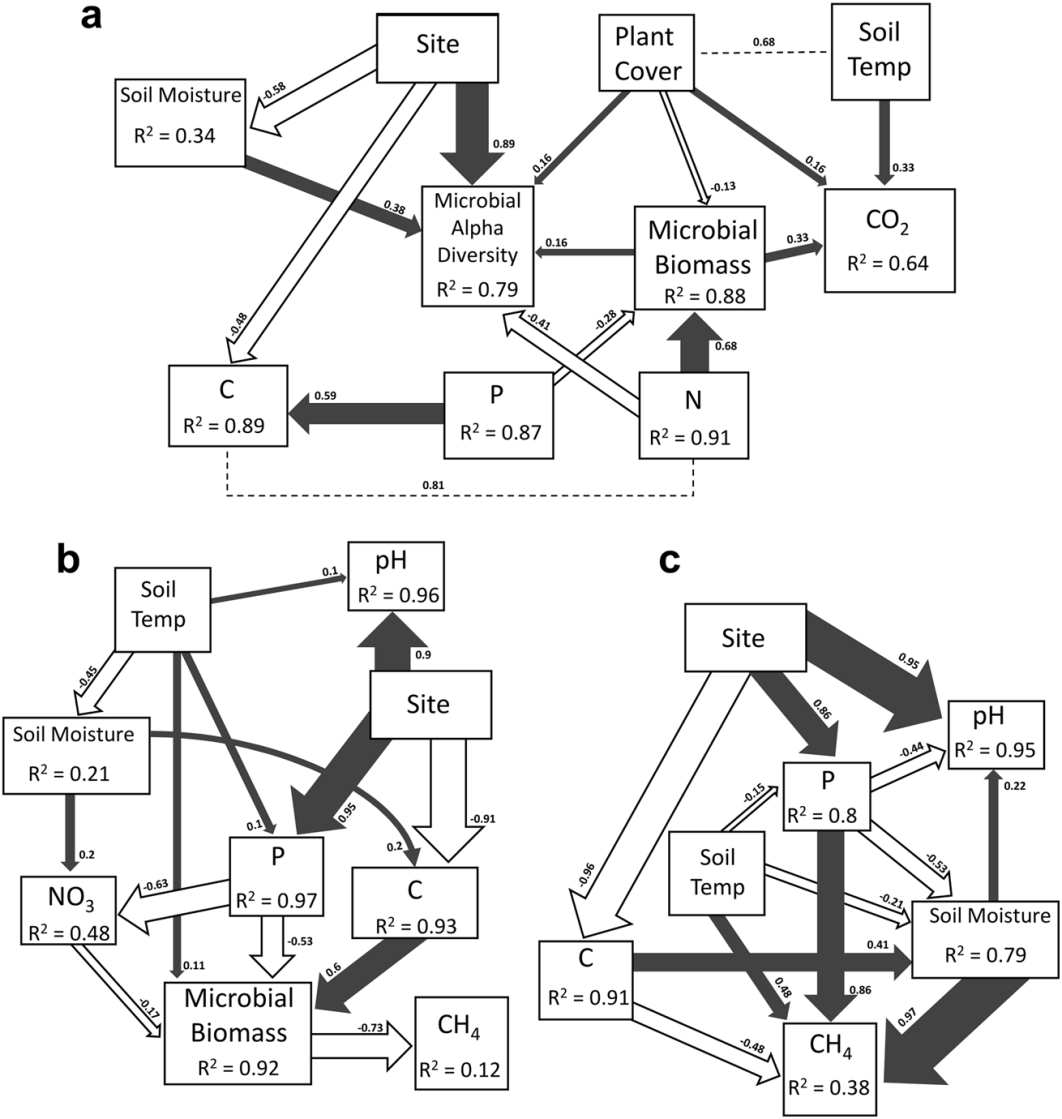
Structural equation modeling showing the relationships among environmental variables and GHG fluxes. **a** Model for total carbon dioxide flux generated from the seasonal data (χ^2^ = 13.355, d.f. = 9, *P* = 0.147, *n* = 84). **b** Model for methane flux generated from seasonal data of switchgrass plots only (χ^2^ = 18.02, d.f. = 17, *P* = 0.388, *n* = 42). **c** Model for methane flux generated from seasonal data of fallow plots only (χ^2^ = 7.131, d.f. = 6, *P* = 0.309, *n* = 42). Dark gray and white arrows represent significant (*p* < 0.05) positive and negative pathways, respectively. Numbers near the pathway arrows indicate the standard path coefficients (*β*). Width of the arrows are proportional to the strength of the relationship. Dashed lines represent residual correlations accounted for in the model. Plant Cover = Switchgrass (positive) or mixed annual grassland plant cover (negative) at the plot; Site = SL (positive) or CL (negative) soil site; CO_2_ = total soil carbon dioxide flux; Soil Temp = soil temperature at a depth of 10 cm for bare soil in degrees Celsius; Soil Moisture = gravimetric per cent soil moisture; P = plant available phosphorus content; Microbial Alpha Diversity = number of observed bacterial species per sample; NO_3_ = nitrate concentrations; CH_4_ = methane flux; and pH = soil pH.

Structural equation modeling (SEM) was used for an in-depth analysis of the direct and indirect effects of environmental drivers on CO_2_ and CH_4_ fluxes. For CO_2_ fluxes (Fig. 7a) the model confirmed the importance of the site effect on soil C and microbial communities, with strong direct effects (based on standardized coefficient) being directed from the site and toward total C (*β* = −0.48, *p* < 0.05) and microbial alpha diversity (*β* = 0.89, *p* < 0.05). Plant available P strongly influenced the levels of C (*β* = 0.59, *p* < 0.01) and microbial biomass in the system (*β* = −0.28, *p* < 0.05). Important variables influencing CO_2_ fluxes included soil temperature (*β* = 0.33, *p* < 0.05), microbial biomass (*β* = 0.33, *p* < 0.05) and plant cover (*β* = 0.16, *p* < 0.05). Microbial biomass appeared mostly dependent on N content (*β* = 0.68, *p* < 0.001) and to a lower extent on P content and the type of plant cover.

In the SEM model for CH_4_ in switchgrass plots (Fig. 7b), the site effect was pronounced and mostly directed toward P levels (*β* = 0.95, *p* < 0.001) and soil C levels (*β* = −0.91, *p* < 0.001). Soil temperature did not have an influence on CH_4_ fluxes directly, it was important in this model via its direct effects on soil moisture (*β* = 0.21, *p* < 0.01), P (*β* = 0.1, *p* < 0.01), and microbial biomass (*β* = 0.11, *p* < 0.05). Microbial biomass was influenced by soil C (*β* = 0.6, *p* < 0.001), plant available P (*β* = −0.53, *p* < 0.001), nitrate level (*β* = −0.17, *p* < 0.01), and soil temperature (*β* = 0.11, *p* < 0.05). Overall, we found that CH_4_ fluxes in the SG plots were directly depended on microbial biomass (*β* = −0.73, *p* < 0.05) suggesting a biological effect to the CH_4_ fluxes. In contrast, the SEM model for CH_4_ fluxes at the FL plots (Fig. 7c) suggested more environmental regulation, as soil moisture (*β* = 0.97, *p* < 0.001), plant available P (*β* = 0.86, *p* < 0.01), soil temperature (*β* = 0.48, *p* < 0.001), and soil C (*β* = −0.48, *p* < 0.05) directly influenced CH_4_ fluxes.

## Discussion

In this study, we examined the differences of soil chemistry, microbial community structure, and GHG fluxes in contrasting soil types with or without switchgrass. As hypothesized, increases in CO_2_ production, and a reduction in alpha and beta microbial community diversity were observed in the CL switchgrass plot. In contrast, at the SL site, we observed an increase in microbial alpha diversity, while beta diversity was reduced; there were no differences in CO_2_ production at this site. However, an increase in topsoil C levels was observed between plots with and without switchgrass for the SL site. Our most striking result is the observation of a systematic and significant reduction of CH_4_ consumption rates, which altered the soil CH_4_ sink capacity in the switchgrass plots. Although the CH_4_ emission rates in this relatively mesic landscape were far lower than those reported for systems with anaerobic, water-logged conditions like peatlands [78] and wetlands [79], CH_4_ emission effects may not be negligible during marginal land transitions to switchgrass row-cropping [40] due to the severity of its global warming potential (28 to 34 times higher than CO_2_). However, comprehensive GHG budgets along with spatially explicit modeling of soil and plant C stocks, should be considered to fully evaluate the effect of large-scale conversion at these prairie sites.

### Soil type dictates the effects that switchgrass has on geochemistry

Our study revealed significant site-level differences in soil C accrual, total soil N levels, and depletion of soil P content after switchgrass establishment. The CL site, with initially higher relative nutrient content, showed little change over time (17 months) for any of the soil geochemical parameters. Reported [12] changes in soil geochemistry by switchgrass cultivation in CL soils were only detected after longer periods of time (over a decade). It is likely that prolonged sampling at our CL site would provide improved assessments of switchgrass-induced soil C changes. We also noted that nitrate content (Fig. S5a) significantly declined with time, over switchgrass establishment, which may suggest assimilation by the plants, microbes, or an increased activity of denitrifying bacteria.

Surface soil C content significantly increased at the SL site (27% higher total C after two growing seasons) over the course of switchgrass establishment. This is consistent with other estimates showing switchgrass systems can increase soil C stocks in a relatively short amount of time [13, 80]. However, we observed significant depletion in soil N and P contents at the SL site with switchgrass over time, though we note that these values were higher than those in the fallow from the beginning of our experiment. One explanation for this observation is that N and P have been taken up into the switchgrass tissue, which could explain the higher belowground root biomass observed in the SL site at lower soil depths. Indeed, the higher plant available P conditions at this site could allow switchgrass to extend deeper into the subsurface soils for water or micronutrient availability and thus support a greater investment of belowground root biomass.

Seasonal sampling of NH_4_-N was not sufficient in explaining large seasonal variations observed over the time course of our experiment. For example, a spike in soil NH_4_ levels (Fig. S5b) was detected during October of 2016 and June of 2017, which could be the signature of episodic N fixation events occurring in switchgrass during/before flowering as reported previously [81]. However, our temporal resolution for this geochemical parameter was not detailed enough to adequately explain these anomalies.

### Microbial community shifts under switchgrass establishment

Microbial community diversity and composition at each site had differential responses to switchgrass establishment. Broadly, alpha diversity measures in the CL switchgrass plot decreased over time and revealed a higher amount of clustering and similarity in the overall community structure compared with the fallow. Analogous to secondary plant successional dynamics, the microbial community at the CL site may be more influenced by the change from short rooted annuals to the monoculture of deep-rooted perennial switchgrass, causing a loss in microbial diversity [82], with a lower number of taxa and more homogeneous community composition. This follows our expectations (hypothesis iii) that switchgrass cultivation would lower microbial alpha and beta diversity. However, for SL, the Shannon index significantly increased over time under switchgrass cultivation and the community composition was altered. This result may be indicative of switchgrass-induced improvements in soil quality (increased soil C concentrations), which led to changes in functionality [83].

Microbial community structure was altered by switchgrass establishment (Table S5) and through time at each of the sites relative to the fallow soil communities. These changes in community structure may reflect different survival strategies that switchgrass may employ in the recruitment of specific taxa to its rhizosphere based on differences between the geochemistry of the two sites. Several previous studies provide evidence that switchgrass can recruit a beneficial microbiome, particularly mycorrhizal fungi, associative N-fixing bacteria, and fungal endophytes [28]. Future investigations into rhizosphere microbiome succession [43–45] during establishment may provide insights into direct plant–microbe interactions that facilitate switchgrass establishment in these nutrient-poor soils.

### Factors controlling soil GHG flux

Contrary to our hypothesis, CO_2_ production was significantly enhanced by switchgrass establishment only at the CL site. Based on previous studies [24, 25], we expected higher root respiration and the potential for deep C mineralization to enhance CO_2_ production at both sites after switchgrass establishment. However, the CL site had an overall higher CO_2_ emission rate during our field monitoring. This response may be mediated by the relatively higher preexisting C content found at this CL site [84], since root biomass levels were estimated to be similar at each site. Our SEM model revealed direct linkages between microbial biomass and CO_2_ production, suggesting that the amount of microbes in the soil play an important role in regulating CO_2_ flux and has long been linked to land usage and environmental variables that influence CO_2_ flux [85]. Overall, our study suggests differential CO_2_ production responses that depend on soil type when under the same land usage (i.e., switchgrass cultivation). Based on our results and those measured for the entire active soil horizon in nearby sites [13], SL marginal sites may provide more sustainable benefits in terms of increased C accrual during switchgrass establishment, as well as reductions in surface CO_2_ fluxes.

With our methane flux monitoring, we showed a significant reduction in CH_4_ consumptions at both sites with switchgrass cultivation. Although CH_4_ emission rates were low and measured at only a few time points within the switchgrass plots, consistently lower CH_4_ consumption rates were observed in the switchgrass plots relative to the fallow plots throughout the experiment. Total CH_4_ consumption rates for switchgrass plots were 47 and 39% lower than in the corresponding fallows for CL and SL, respectively. During the period of 2008–2017, global emissions from both agriculture and waste sources were estimated to be 206 Tg CH_4_ y^−1^, which represents 56% of the total anthropogenic emissions [86]. Recent estimates for 2018 and 2019 have shown an increase in atmospheric methane by 8.5 and 10.7 ppb [87], respectively, prompting the need for future mitigation, since CH_4_ has 28 to 34 times the global warming potential (GWP) over a 100-year time horizon than CO_2_ [88]. Based on our results, CH_4_ flux profiles of each site may be altered by an estimated 0.48 kg ha^−1^ yr^−1^ when cultivated with switchgrass. Methane flux from our SEM models was also found to be correlated with microbial biomass, but only in the SG plots, suggesting a potential biological effect of switchgrass on the microbial community, which may have promoted the growth of key community members that can alter methane emissions (i.e., methanogens or methanotrophs). However, our analyses of community composition could not support further associations between CH_4_ flux and microbial community members. In contrast to CO_2_ and CH_4_ fluxes, we did not observe any significant effect between soil type and plant cover type (switchgrass vs fallow) on N_2_O fluxes in these marginal, sustainably managed (no fertilizer) soils.

## Conclusion

Overall, total soil C levels increased by 27% following switchgrass planting and 17 months of monitoring in our SL site, but remained unchanged in our CL site. Total CO_2_ production was significantly affected under switchgrass at the CL site but not at the SL site. The annual CH_4_ consumption was reduced by 39 to 47% under switchgrass, implying that methane fluxes should be accounted for in C budgets to reach a sustainable cultivation of switchgrass. Switchgrass establishment had a significant influence on the microbial community composition over time and homogenized soil microbiota profiles at both sites. Considerations of soil type and nutrient conditions should be factors in the selection of future sites for sustainable large-scale bioenergy cultivation in order to meet objectives for terrestrial C sequestration and improved soil fertility.

## Supplementary information

Figure S1

Figure S2

Figure S3

Figure S4

Figure S5

Figure S6

Table S1

Table S2

Table S3

Table S4

Table S5

Table S6
