# Supplementary figures and images for: Conversion of marginal land into switchgrass conditionally accrues soil carbon but reduces methane consumption
Source: ISME J. 2021 Jul 1;16(1):10–25. doi: 10.1038/s41396-021-00916-y (PMC8692414; doi:10.1038/s41396-021-00916-y)

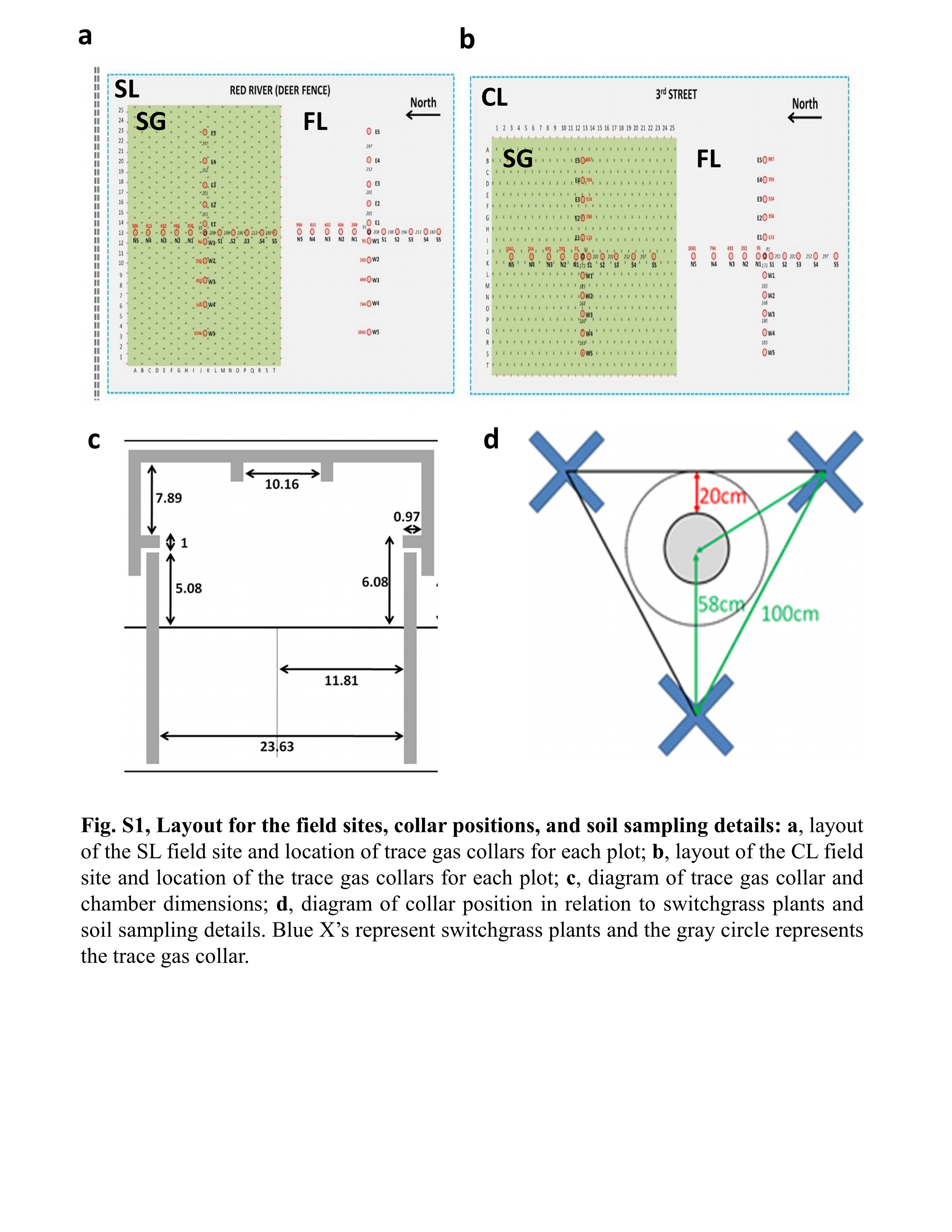

Supplement: Supplementary file 1 — Figure S1 [file 41396_2021_916_MOESM1_ESM.tif]

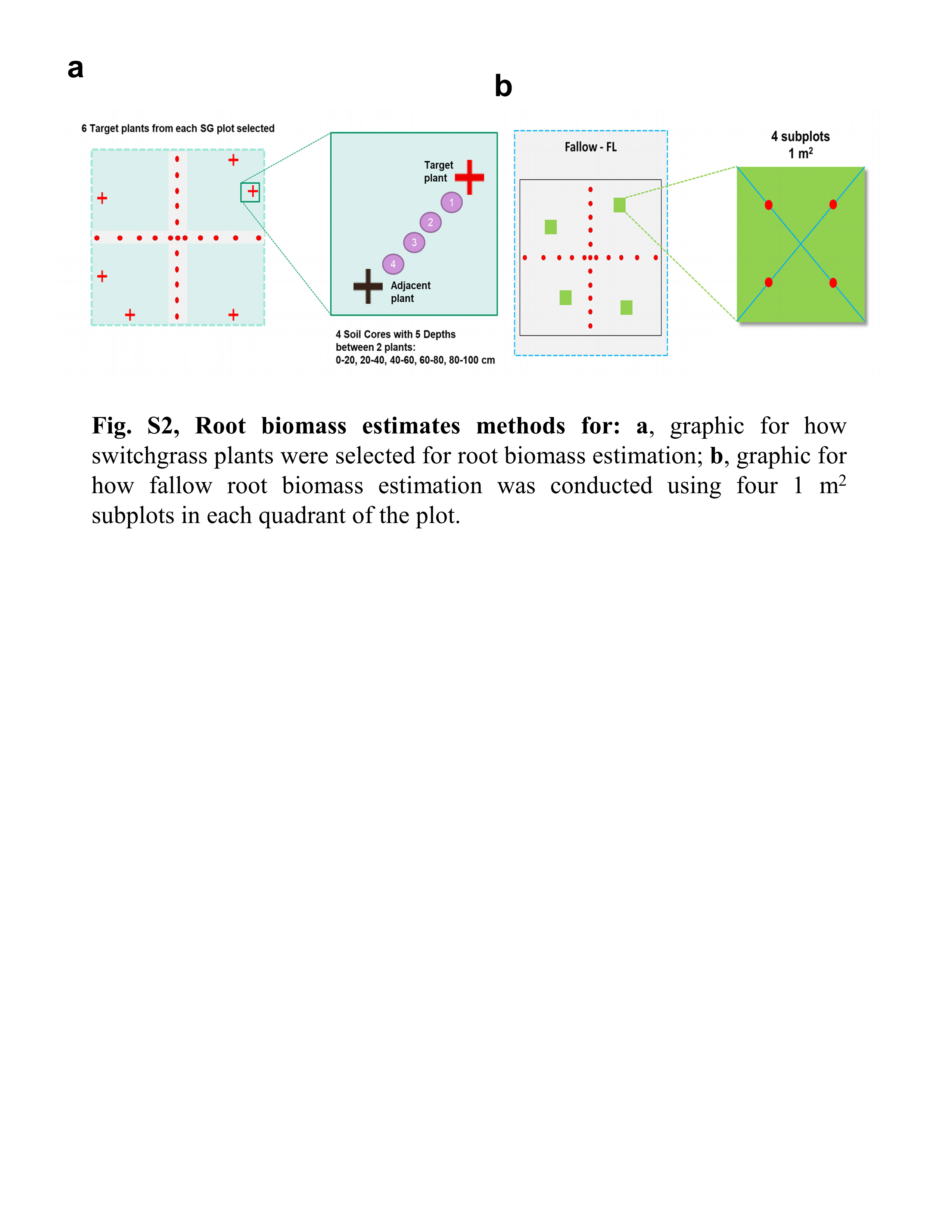

Supplement: Supplementary file 2 — Figure S2 [file 41396_2021_916_MOESM2_ESM.tif]

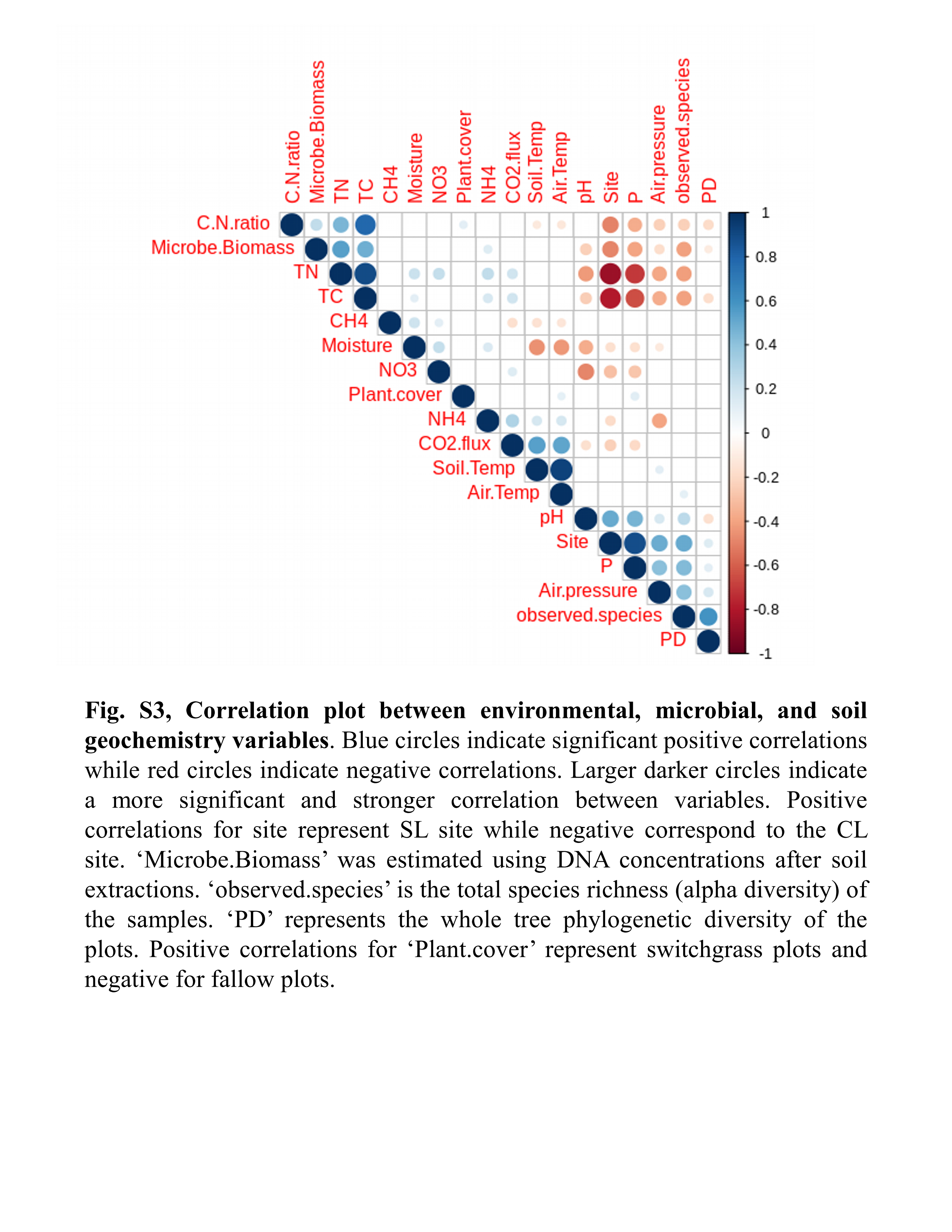

Supplement: Supplementary file 3 — Figure S3 [file 41396_2021_916_MOESM3_ESM.tif]

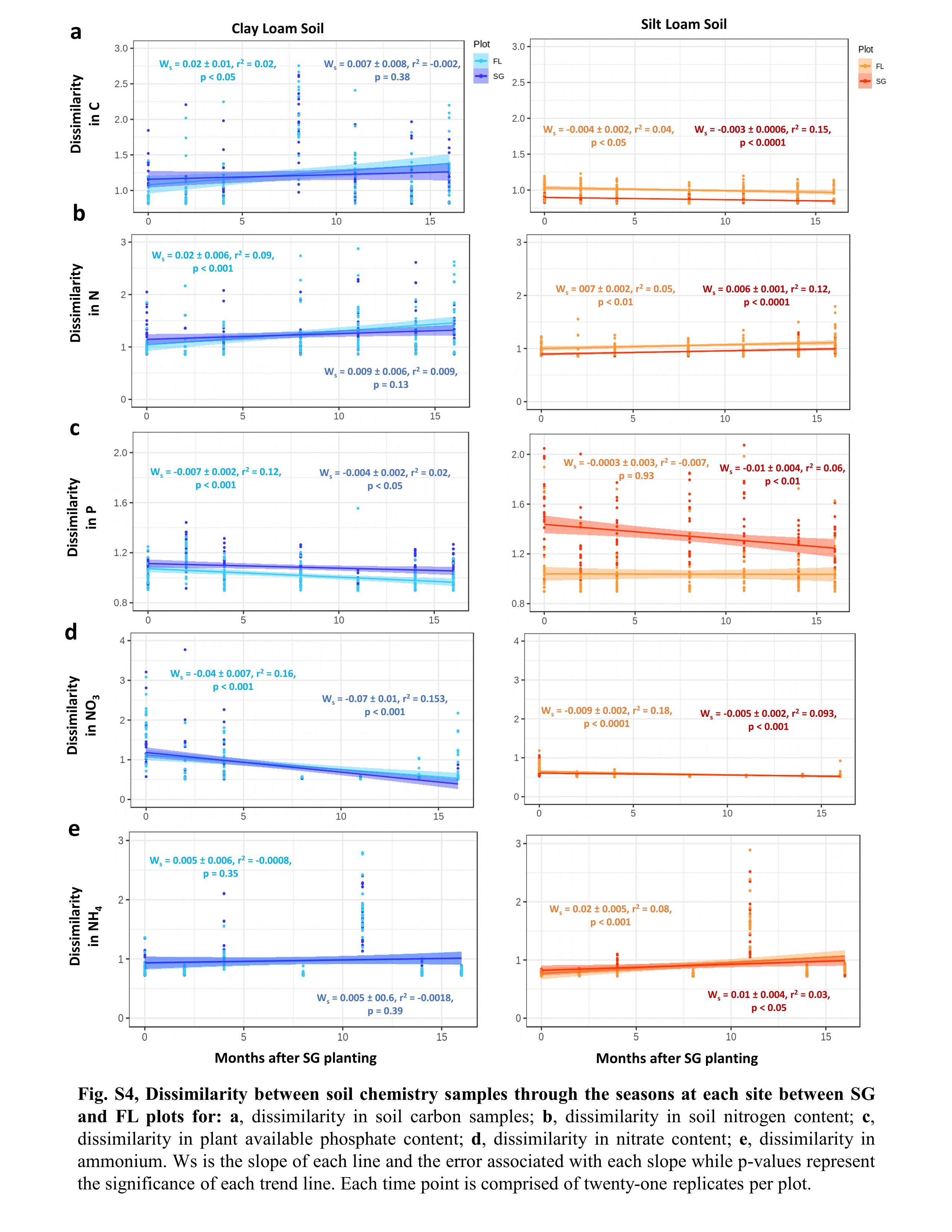

Supplement: Supplementary file 4 — Figure S4 [file 41396_2021_916_MOESM4_ESM.tif]

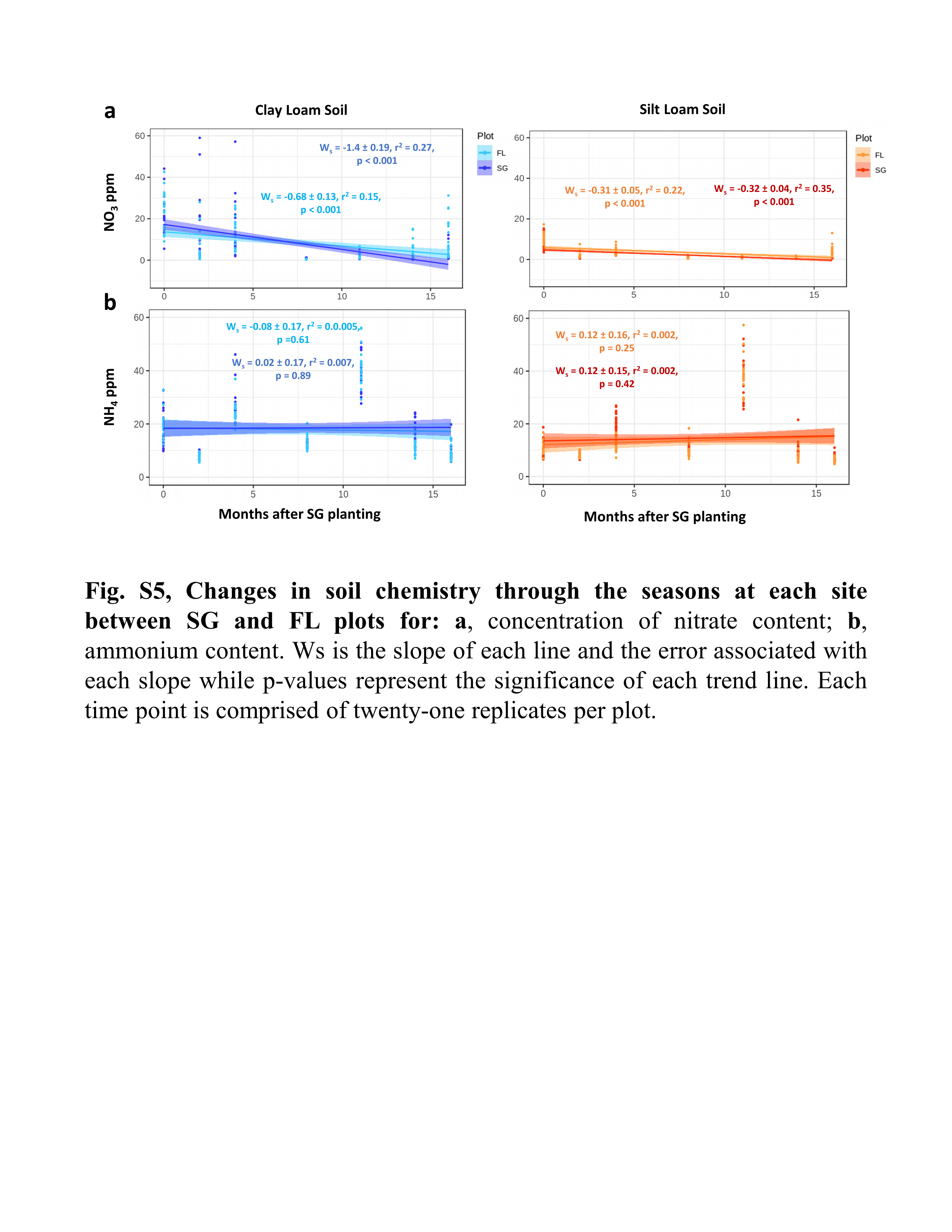

Supplement: Supplementary file 5 — Figure S5 [file 41396_2021_916_MOESM5_ESM.tif]

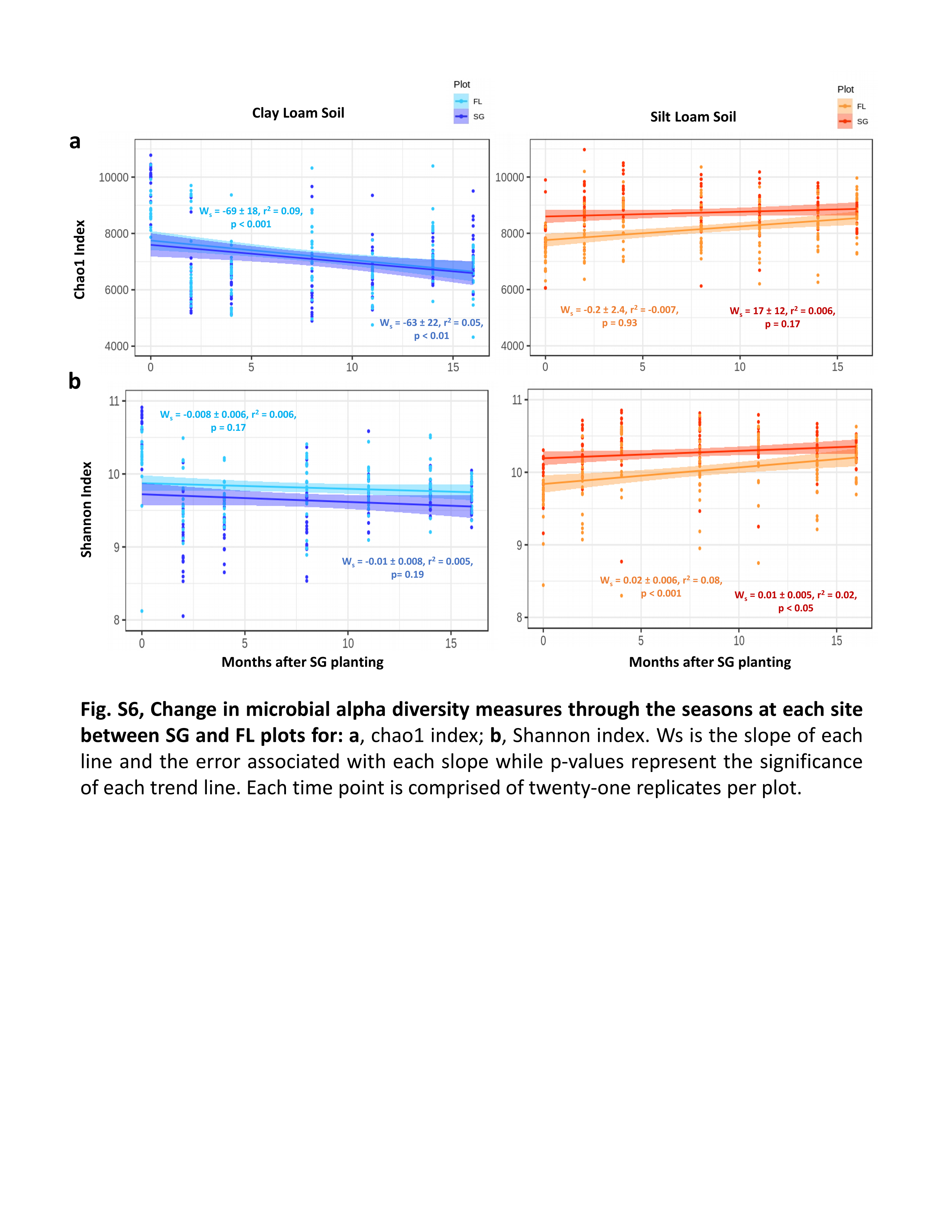

Supplement: Supplementary file 6 — Figure S6 [file 41396_2021_916_MOESM6_ESM.tif]
